# Fair enough: A sideways look

**DOI:** 10.1016/j.patter.2021.100331

**Published:** 2021-08-13

**Authors:** Jeremy G. Frey

**Affiliations:** 1 School of Chemistry, University of Southampton, Southampton, SO17 1BJ, UK

## Abstract

Even when we all agree to make data usefully available with our publications, the problem remains of how to do this in a reasonable and sensible manner. Just what do we need to do? I suggest a change of perspective: what would you wish other authors had done to make their data easily useful to us?

## Main text

When asked by my colleagues, “What exactly is needed to comply with data management policies and to make my data FAIR [findable, accessible, interoperable, and reusable)?”^1^ my short answer is in line with the Golden Rule, “Do unto others how you would wish them to do unto you.”

My longer reply is along these lines: if you read about an interesting experiment in a paper in your research area, and you want to use the result and incorporate the experiment in your own work, what information, what details would you need the authors to have provided? Go ahead and provide that level of detail.

It might be better even to suggest that the colleague reflects on when they need information from a paper not in their immediate area, where they are less acquainted with all the fine details of the research, what information would they need (hope) to find?

The next level of this spiral argument would be to think about what details would a new graduate student working in the area need to be able to locate the paper in the first place and then extract the necessary information to be able to (correctly) use the outputs of your research (and of course acknowledge the source).

But at what point should this stop? Does the paper need a whole undergraduate module to be followed first before it can be understood? Probably, and perhaps we should provide a link to potential courses as part of the background? But surely this is taking things to an extreme, unless the research is in a novel area, when it might be necessary and indeed worthwhile to go to this amount of work to ensure your research does have the opportunity to create impact in the wider(est) community.

How to decide when is the appropriate point to halt this spiral where the contextual information could easily overwhelm the new results? The Royal Society Report on Science and an Open Enterprise^2^ highlighted the concept of “intelligently open” to reflect the necessary interplay between the author and the reader, to reflect open communication. Without this concept or something similar, one could either ignore the need to provide the necessary context, or in a revision of the famous English oxymoron “fair unfairly kept them falsely true,” by too great an attention to the detail of the letter of the data management plans, one could miss the whole aim of the open science ideal.

At conferences over the last few years, I have noticed that industry interest in data sharing (and thus uptake of FAIR) has been much greater than in the academic world. Initially this might seem surprising—commercial interests are usually viewed and benefit from closely guarding access to data and information, while academia is about contributing to the scientific literature. However, industry is also often about a team effort to achieve and aim—industries need to work together, and any barrier to integrating the team efforts has a direct commercial cost. This makes very clear the benefit of easy exchange of data and the costs of failing to do so. In contrast, the academic environment can drive (though not necessarily) a very personal perspective. Not only should this provide an example to universities but highlight the need to train the next generation of researchers to take their place in these industrial teams.

Having worked out what data to present to one’s colleagues to ensure your work is understandable and repeatable, then one of the most difficult things to establish is whether the format (file format) we use for our data are sufficiently universally understood so that they can in fact make use of the data. The FAIRSharing project^3^ is a useful resource in this regard.

The ubiquitous CSV file provides a simple data management example that was brought home to me when teaching Python (I am usually an R programmer). It is great that the file format has the ability (not always used) to give sensible names to each column of data, and this clearly helps the use and re-use of the data. But when teaching, I realized that I was falling into the trap of “do as I say not what I do”—all data files should have at least a title within them so that even when the file name is changed, it will still be clear what the data in the file is about. What is the catch? Well, all the obvious routines for reading CSV files don’t consider the possibility of a title and comments as well as column headers. This is easy to fix in a program but changes the standard.

What would I like to see happen? What we need to achieve is an enhancement to the research culture, at the start of the UK e-Science program we suggested in Publication @ Source^4^, ^5^, ^6^ that the mindset of those undertaking research should, right from the start, have the view that others will use their work and so plan, collect, and record with this in mind. I was inspired by the 2014 BBC Reith Lectures given by Dr. Atul Gawande^7^ in discussing complex medical systems and how to ensure high-quality outcomes. Providing and explaining incentives (encourage) and enforcement (Medieval punishment) to push adoption only go so far; proper design is critical in imbedding a culture change. In his book *The Checklist Manifesto*,^8^ he demonstrates the importance of having a process for whatever you are doing to ensure these quality outcomes.

In many ways my own areas of research in physical sciences are simple. Once a decision to publish is made, there is little reason not to make all the data available, and indeed, doing so will help with assigning credit where credit is due, something that has often been overlooked. However, in my wider work with the digital economy, with the need to obtain ethical approval, I am well aware that in many research areas, for example those involving people, even if only expressing simply personal views, exposing data, even with some degree of anonymization, can be very problematic for the communities involved.

As part of the international standards work, IUPAC is working on recommendations for FAIR Chemistry (see https://www.fairsfair.eu/ and specifically https://www.fairsfair.eu/recommendations-practice-support-fair-data-principles and https://www.go-fair.org/fair-principles/), because while high-level guidelines do exist (for examples see https://iupac.org/wp-content/uploads/2020/10/CPCDS_Shorts_JulyAug2020.pdf and https://iupac.org/projects/project-details/?project_nr=2019-031-1-024), useful applicable guidelines at a disciplinary level are lacking but are essential to the digital transformation of IUPAC and chemistry as a whole.^9^^,^^10^ We need to start from professional understanding of the necessity to collaborate and communicate, and following Gawande, we need clear processes. Reflecting on what we would want others to do for us, is enormously helpful in thinking what these processes should be.

Have I always followed these ideals? No, but I am trying!

## Web resources

Committee on Publications and Cheminformatics Data Standards August 2 Update, https://iupac.org/wp-content/uploads/2020/10/CPCDS_Shorts_JulyAug2020.pdf

FAIR Principles, https://www.go-fair.org/fair-principles/

FAIRsFAIR, https://www.fairsfair.eu/

International Union of Pure and Applied Chemistry, https://iupac.org/projects/project-details/?project_nr=2019-031-1-024

Recommendations on practice to support FAIR data principles, https://www.fairsfair.eu/recommendations-practice-support-fair-data-principles
